# Supplementary figures and images for: Alternative dietary protein and water temperature influence the skin and gut microbial communities of yellowtail kingfish (Seriola lalandi)
Source: PeerJ. 2020 Mar 19;8:e8705. doi: 10.7717/peerj.8705 (PMC7085898; doi:10.7717/peerj.8705)

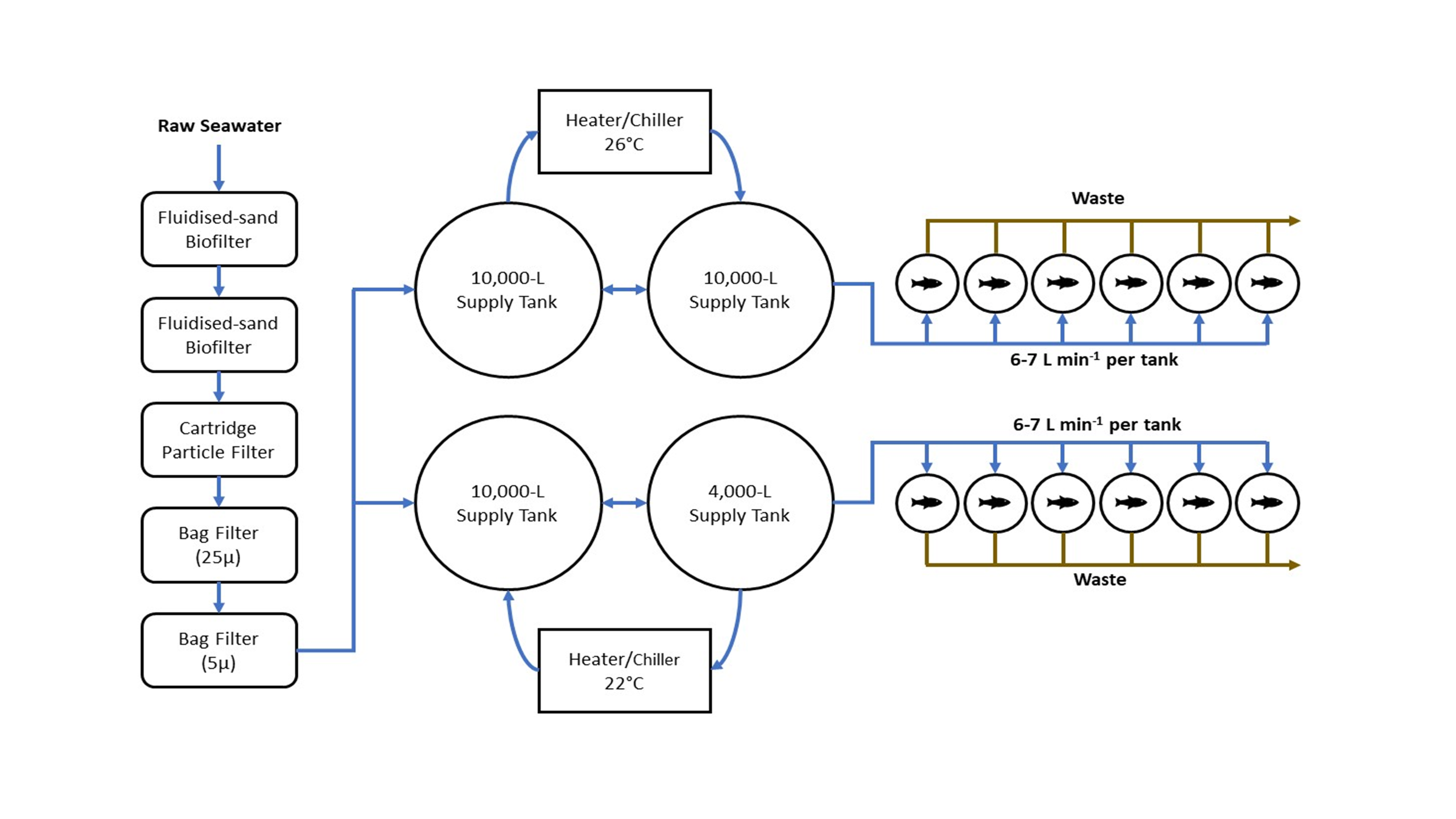

Supplement: Supplemental Information 1 [file peerj-08-8705-s001.png]

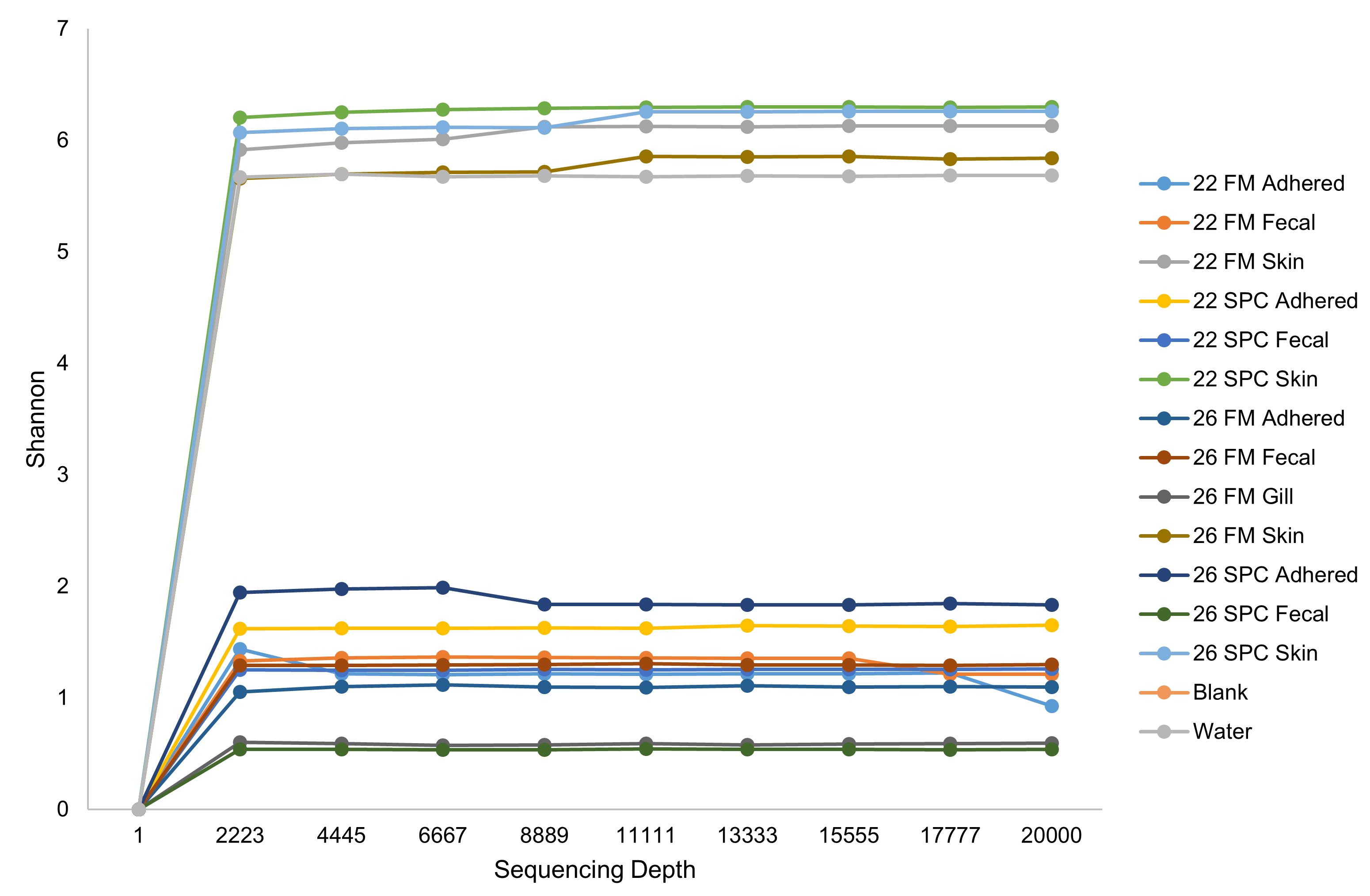

Supplement: Supplemental Information 2 — Rarefaction curves describing bacterial diversity (Shannon) at different sequencing depths for each treatment within each body site [file peerj-08-8705-s002.png]

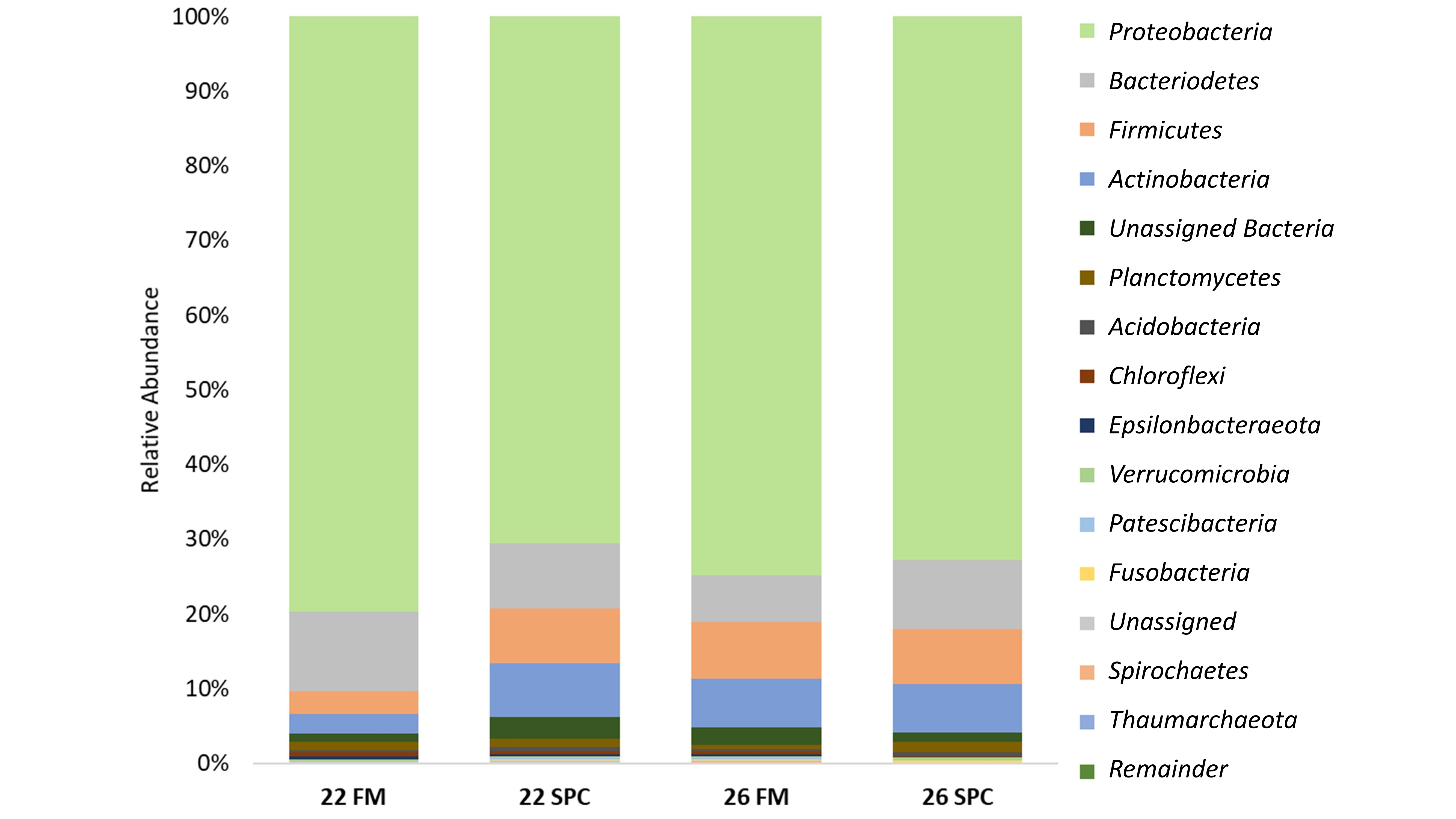

Supplement: Supplemental Information 3 — Mean relative abundance of OTUs in each treatment grouped by phylum of final skin mucosal samples. Phyla comprising less than 0.2% grouped under remainder. [file peerj-08-8705-s003.png]
