# Supplementary material for: Alternative dietary protein and water temperature influence the skin and gut microbial communities of yellowtail kingfish (Seriola lalandi)
Source: PeerJ. 2020 Mar 19;8:e8705. doi: 10.7717/peerj.8705 (PMC7085898; doi:10.7717/peerj.8705)
Supplement: Supplemental Information 4 — aValues are given in g kg−1 dry matter unless otherwise stated. [file peerj-08-8705-s004.docx]

|  | **FM** | **SPC** |
| --- | --- | --- |
| **Ingredients** |  |  |
| Fishmeal | 682.4 | 475.1 |
| Soy Protein Concentrate | - | 300.0 |
| Wheat flour | 270.0 | 188.0 |
| Fish oil | 35.0 | 24.4 |
| Choline chloride (70%) | 6.0 | 6.0 |
| Vitamin C (Stay-C 35®) | 0.6 | 0.6 |
| Vitamin / mineral premix | 6.0 | 6.0 |
| Total dry matter | 1,000.0 | 1,000.0 |
|  |  |  |
| **Nutrient composition** |  |  |
| Nitrogen | 91.3 | 93.6 |
| Crude protein | 570.8 | 585.2 |
| Ash | 125.0 | 109.8 |
| Lipid | 83.7 | 61.2 |
| NFE | 220.5 | 243.8 |
| Gross energy (MJ kg^-1^) | 20.8 | 20.8 |
|  |  |  |
| **Amino acid composition** |  |  |
| Alanine | 34.7 | 31.4 |
| Arginine | 26.4 | 31.2 |
| Aspartic acid (+ asparagine) | 52.8 | 61.1 |
| Cysteine | 5.8 | 5.3 |
| Glutamic acid (+ glutamine) | 83.3 | 94.6 |
| Glycine | 38.9 | 33.9 |
| Histidine | 9.8 | 9.6 |
| Isoleucine | 19.9 | 21.1 |
| Leucine | 39.7 | 40.9 |
| Lysine | 35.2 | 36.5 |
| Methionine | 13.1 | 12.8 |
| Phenylalanine | 23.9 | 25.6 |
| Proline | 26.6 | 28.2 |
| Serine | 28.1 | 30.5 |
| Taurine | 13.6 | 12.5 |
| Threonine | 24.0 | 24.7 |
| Tyrosine | 18.8 | 21.1 |
| Valine | 26.4 | 26.8 |
| Total reported amino acids + taurine | 520.9 | 547.9 |
