# Supplementary material for: Alternative dietary protein and water temperature influence the skin and gut microbial communities of yellowtail kingfish (Seriola lalandi)
Source: PeerJ. 2020 Mar 19;8:e8705. doi: 10.7717/peerj.8705 (PMC7085898; doi:10.7717/peerj.8705)
Supplement: Supplemental Information 5 [file peerj-08-8705-s005.docx]

| **Water Parameter** |  | **22 FM** |  | **22 SPC** |  | **26 FM** |  | **26 SPC** |
| --- | --- | --- | --- | --- | --- | --- | --- | --- |
| Temperature (°C) |  | 22.1 ± 0.3 |  | 22.1 ± 0.3 |  | 26.1 ± 0.2 |  | 26 ± 0.2 |
| pH |  | 8.6 ± 0.1 |  | 8.6 ± 0.1 |  | 8.6 ± 0.2 |  | 8.6 ± 0.1 |
| Dissolved Oxygen (%) |  | 101.4 ± 4.4 |  | 102 ± 4.7 |  | 99.4 ± 5.5 |  | 98.6 ± 5.8 |
| Salinity (%) |  | 3.7 ± 0.04 |  | 3.7 ± 0.05 |  | 3.7 ± 0.05 |  | 3.7 ± 0.04 |
