# Supplementary material for: Alternative dietary protein and water temperature influence the skin and gut microbial communities of yellowtail kingfish (Seriola lalandi)
Source: PeerJ. 2020 Mar 19;8:e8705. doi: 10.7717/peerj.8705 (PMC7085898; doi:10.7717/peerj.8705)
Supplement: Supplemental Information 6 — aPairwise PERMANOVA with 999 permutations was performed on a Bray–Curtis dissimilarity matrix. [file peerj-08-8705-s006.docx]

| **Body site** | **R2** | **p** |
| --- | --- | --- |
| Skin | 0.079 | 0.181 |
| Digesta | 0.065 | 0.286 |
| Gut Mucosa | 0.894 | 0.387 |
