# Supplementary material for: Alternative dietary protein and water temperature influence the skin and gut microbial communities of yellowtail kingfish (Seriola lalandi)
Source: PeerJ. 2020 Mar 19;8:e8705. doi: 10.7717/peerj.8705 (PMC7085898; doi:10.7717/peerj.8705)
Supplement: Supplemental Information 7 — aPairwise PERMANOVA with 999 permutations was performed on a Bray–Curtis dissimilarity matrix. [file peerj-08-8705-s007.docx]

| **Tanks compared** | **Treatment** | **Bodysite** |  | **R2** |  | **pvalue** |
| --- | --- | --- | --- | --- | --- | --- |
| 2 Vs 4 | 22 Fishmeal | Adhered |  | 0.15 |  | 0.60 |
| 2 Vs 5 | 22 Fishmeal | Adhered |  | 0.16 |  | 0.58 |
| 4 Vs 5 | 22 Fishmeal | Adhered |  | 0.17 |  | 0.74 |
| 2 Vs 4 | 22 Fishmeal | Fecal |  | 0.28 |  | 0.08 |
| 2 Vs 5 | 22 Fishmeal | Fecal |  | 0.44 |  | 0.11 |
| 4 Vs 5 | 22 Fishmeal | Fecal |  | 0.56 |  | 0.09 |
| 2 Vs 4 | 22 Fishmeal | Skin |  | 0.1 |  | 0.66 |
| 2 Vs 5 | 22 Fishmeal | Skin |  | 0.57 |  | 0.04 |
| 4 Vs 5 | 22 Fishmeal | Skin |  | 0.54 |  | 0.04 |
| 1 Vs 3 | 22 SPC | Adhered |  | 0.36 |  | 0.58 |
| 1 Vs 6 | 22 SPC | Adhered |  | 0.27 |  | 0.49 |
| 3 Vs 6 | 22 SPC | Adhered |  | 0.09 |  | 0.91 |
| 1 Vs 3 | 22 SPC | Fecal |  | 0.24 |  | 0.18 |
| 1 Vs 6 | 22 SPC | Fecal |  | 0.19 |  | 0.12 |
| 3 Vs 6 | 22 SPC | Fecal |  | 0.21 |  | 0.23 |
| 1 Vs 3 | 22 SPC | Skin |  | 0.6 |  | 0.11 |
| 1 Vs 6 | 22 SPC | Skin |  | 0.48 |  | 0.15 |
| 3 Vs 6 | 22 SPC | Skin |  | 0.2 |  | 0.13 |
| 10 Vs 11 | 26 Fishmeal | Adhered |  | 0.16 |  | 0.66 |
| 10 Vs 9 | 26 Fishmeal | Adhered |  | 0.12 |  | 0.90 |
| 11 Vs 9 | 26 Fishmeal | Adhered |  | 0.37 |  | 0.72 |
| 10 Vs 11 | 26 Fishmeal | Fecal |  | 0.19 |  | 0.48 |
| 10 Vs 9 | 26 Fishmeal | Fecal |  | 0.21 |  | 0.24 |
| 11 Vs 9 | 26 Fishmeal | Fecal |  | 0.18 |  | 0.38 |
| 10 Vs 11 | 26 Fishmeal | Skin |  | 0.16 |  | 0.56 |
| 10 Vs 9 | 26 Fishmeal | Skin |  | 0.3 |  | 0.27 |
| 11 Vs 9 | 26 Fishmeal | Skin |  | 0.26 |  | 0.08 |
| 12 Vs 7 | 26 SPC | Adhered |  | 0.27 |  | 0.38 |
| 12 Vs 8 | 26 SPC | Adhered |  | 0.4 |  | 0.15 |
| 7 Vs 8 | 26 SPC | Adhered |  | 0.16 |  | 1.00 |
| 12 Vs 7 | 26 SPC | Fecal |  | 0.15 |  | 0.60 |
| 12 Vs 8 | 26 SPC | Fecal |  | 0.18 |  | 0.25 |
| 7 Vs 8 | 26 SPC | Fecal |  | 0.2 |  | 0.15 |
| 12 Vs 7 | 26 SPC | Skin |  | 0.34 |  | 0.38 |
| 12 Vs 8 | 26 SPC | Skin |  | 0.13 |  | 0.91 |
| 7 Vs 8 | 26 SPC | Skin |  | 0.29 |  | 0.38 |
