# Supplementary material for: Alternative dietary protein and water temperature influence the skin and gut microbial communities of yellowtail kingfish (Seriola lalandi)
Source: PeerJ. 2020 Mar 19;8:e8705. doi: 10.7717/peerj.8705 (PMC7085898; doi:10.7717/peerj.8705)
Supplement: Supplemental Information 8 [file peerj-08-8705-s008.docx]

| **Sample Name** | **Protein Alignment** | **Accession** | **Identity** |
| --- | --- | --- | --- |
| 124 | Thermolabile hemolysin [*Photobacterium damselae*] | WP_106261769 | 100% |
| 180 | Thermolabile hemolysin [*Photobacterium damselae*] | WP_065172159 | 100% |
| 188 | Thermolabile hemolysin [*Photobacterium damselae*] | WP_106261769 | 100% |
