# Supplementary material for: Alternative dietary protein and water temperature influence the skin and gut microbial communities of yellowtail kingfish (Seriola lalandi)
Source: PeerJ. 2020 Mar 19;8:e8705. doi: 10.7717/peerj.8705 (PMC7085898; doi:10.7717/peerj.8705)
Supplement: Supplemental Information 9 [file peerj-08-8705-s009.docx]

| **Sample Name** | **Alignment** | **Accession** | **Identity** |
| --- | --- | --- | --- |
| 124 | *Photobacterium damselae* subsp. *damselae* strain KC-Na-1 | CP021151 | 99.8% |
|  | *Photobacterium damselae* strain Phdp Wu-1 | CP018297 | 99.7% |
|  | *Photobacterium damselae* subsp. *piscicida* DNA | AP018045 | 99.8% |
| 180 | *Photobacterium damselae* subsp. *damselae* strain KC-Na-1 | CP021151 | 95.80% |
|  | *Photobacterium damselae* strain Phdp Wu-1 | CP018297 | 95.80% |
|  | *Photobacterium damselae* subsp. *piscicida* DNA | AP018045 | 96% |
| 188 | *Photobacterium damselae* subsp. *damselae* strain KC-Na-1 | CP021151 | 99.7% |
|  | *Photobacterium damselae* strain Phdp Wu-1 | CP018297 | 99.7% |
|  | *Photobacterium damselae* subsp. *piscicida* DNA | AP018045 | 99.8% |
