# Supplementary material for: Alternative dietary protein and water temperature influence the skin and gut microbial communities of yellowtail kingfish (Seriola lalandi)
Source: PeerJ. 2020 Mar 19;8:e8705. doi: 10.7717/peerj.8705 (PMC7085898; doi:10.7717/peerj.8705)
Supplement: Supplemental Information 11 — aPairwise PERMANOVA with 999 permutations was performed on a Bray–Curtis dissimilarity matrix. [file peerj-08-8705-s011.docx]

| **Control** | **Treatment** | **Differ by** | **R^2^** | **p** |
| --- | --- | --- | --- | --- |
| 22 FM | 22 SPC | Diet | 0.105 | 0.412 |
| 22 FM | 26 FM | Temp | 0.115 | 0.689 |
| 22 FM | 26 SPC | Diet + Temp | 0.135 | 0.516 |
